# Supplementary material for: A Novel Method to Assess Antimicrobial Susceptibility in Commensal Oropharyngeal Neisseria—A Pilot Study
Source: Antibiotics (Basel). 2022 Jan 13;11(1):100. doi: 10.3390/antibiotics11010100 (PMC8772996; doi:10.3390/antibiotics11010100)
Supplement: Supplementary file 1 [file antibiotics-11-00100-s001.zip › antibiotics-1530306-supplementary.pdf]

Supplemental Table S1. Bacterial species isolated using MALDI-TOF-MS from paired oral rinse and oropharyngeal swab samples present on the azithromycin-containing plate (2 μg/mL) and the plate without azithromycin.

Participant	Oral Rinse sample on plate -AZM	Oral Rinse sample on plate +AZM	Swab sample on plate -AZM	Swab sample on plate +AZM
1	*Neisseria subflava*<br>*Neisseria macacae*	*Neisseria subflava*<br>*Neisseria spp.* - Most likely *N. macacae*	*Neisseria subflava*<br>*Neisseria macacae*	*Neisseria subflava*<br>*Neisseria spp.* - Most likely *N. macacae*
2	*Neisseria subflava*<br>*Neisseria macacae*	*Neisseria subflava*<br>*Neisseria bacilliformis*	*Neisseria subflava*<br>*Neisseria macacae*	
3	*Neisseria subflava*<br>*Neisseria macacae*	*Neisseria subflava*<br>*Neisseria macacae*	*Neisseria subflava*<br>*Neisseria macacae*	*Neisseria subflava*
4	*Neisseria subflava*<br>*Neisseria macacae*	*Neisseria subflava*<br>*Neisseria macacae*	*Neisseria subflava*<br>*Neisseria macacae*	*Neisseria subflava*<br>*Neisseria spp.* - Most likely *N. macacae*
5	*Neisseria subflava*<br>*Neisseria elongata*	*Neisseria subflava*	*Neisseria subflava*<br>*Neisseria elongata*	*Neisseria subflava*
6	*Neisseria subflava*<br>*Neisseria macacae*	*Neisseria subflava*<br>*Neisseria spp.* - Most likely *N. macacae*	*Neisseria subflava*<br>*Neisseria macacae*	*Neisseria subflava*<br>*Neisseria spp.* - Most likely *N. macacae*
7	*Neisseria subflava*<br>*Neisseria elongata*<br>*Moraxella spp/*	*Neisseria subflava*<br>*Neisseria macacae*	*Neisseria subflava*<br>*Neisseria macacae*	*Neisseria subflava*<br>*Neisseria macacae*
8	*Neisseria subflava*	*Neisseria subflava*		*Neisseria subflava*
9	*Neisseria subflava*	*Neisseria subflava*	*Neisseria subflava*	*Neisseria subflava*
10	*Neisseria subflava*<br>*Neisseria oralis*<br>*Brevundimonas spp.*	*Neisseria subflava*<br>*Brevundimonas spp.*	*Neisseria subflava*<br>*Brevundimonas spp.*	*Neisseria subflava*

AZM= azithromycin, *spp.*=species, *Neisseria macacae= Neisseria macacae and Neisseria mucosa, Neisseria subflava= Neisseria subflava/Neisseria flavescens/Neisseria perflava*

Supplemental Table S2. Absolute number of colonies counted from diluted oral rinse and swab samples on the LBVT-SNR plates with and without azithromycin.

Participant	Condition	Dilution 10^{-1}	Dilution 10^{-2}	Dilution 10^{-3}
1	Swab	138	46	<20
	Swab + AZM	96	<20	<20
	Oral rinse	>200	205	<20
	Oral rinse + AZM	>200	95	<20
2	Swab	<20	<20	<20
	Swab + AZM	<20	<20	<20
	Oral rinse	>200	68	<20
	Oral rinse + AZM	48	<20	<20
3	Swab	128	<20	<20
	Swab + AZM	26	<20	<20
	Oral rinse	>200	>200	164
	Oral rinse + AZM	>200	>200	82
4	Swab	>200	>200	40
	Swab + AZM	>200	>200	75
	Oral rinse	>200	>200	87
	Oral rinse + AZM	>200	>200	69
5	Swab	>200	>200	149
	Swab + AZM	>200	>200	34
	Oral rinse	>200	60	<20
	Oral rinse + AZM	>200	66	<20
6	Swab	>200	>200	154
	Swab + AZM	>200	>200	36
	Oral rinse	>200	>200	145
	Oral rinse + AZM	>200	>200	156
7	Swab	>200	>200	50
	Swab + AZM	>200	>200	35
	Oral rinse	>200	>200	62
	Oral rinse + AZM	>200	>200	37
8	Swab	<20	<20	<20
	Swab + AZM	<20	<20	<20
	Oral rinse	>200	109	<20
	Oral rinse + AZM	>200	38	<20
	Swab	>200	61	<20
9	Swab + AZM	>200	36	<20
9	Oral rinse	>200	169	<20
	Oral rinse + AZM	>200	116	<20
10	Swab	>200	>200	29
	Swab + AZM	>200	>200	22
	Oral rinse	>200	>200 (376)	*
	Oral rinse + AZM	>200	>200 (408)	65

AZM= azithromycin, *= Culture not evenly distributed, uncountable
